# Supplementary material for: Exercise can improve sleep quality: a systematic review and meta-analysis
Source: PeerJ. 2018 Jul 11;6:e5172. doi: 10.7717/peerj.5172 (PMC6045928; doi:10.7717/peerj.5172)
Supplement: Article S1 — The contribution that the meta-analysis makes to knowledge in light of previously published related reports, including other meta-analyses and systematic reviews. [file peerj-06-5172-s026.docx]

**1. The rationale for conducting the meta-analysis**

Exercise is a nonpharmacological therapy for insomnia, is readily available, and costs less than other nonpharmacological treatments for insomnia; notably, its effects depend upon exercise type and evaluation methodology (Youngstedt, O'Connor & Dishman, 1997; Driver & Taylor, 2000; Youngstedt, 2005). Recent randomized controlled trials (RCTs) have confirmed that exercise can improve sleep quality, sleep onset latency, total sleep time, sleep efficiency, and insomnia severity (Passos et al., 2010; Reid et al., 2010; Hartescu, Morgan & Stevinson, 2015). The beneficial effect of exercise on sleep may be explained by the interaction between the circadian rhythm and metabolic, immune, thermoregulatory, vascular, mood, and endocrine effects (Chennaoui et al., 2015).

The fifth edition of the Diagnostic and Statistical Manual of Mental Disorders (DSM-5) and the third edition of the International Classification of Sleep Disorders (ICSD-3) made major revisions to their definitions of insomnia. The DSM-5 and ICSD-3 abolished the distinction between primary and secondary insomnia. The revision was based on the findings that insomnia: 1) often accompanies another disease; 2) is preceded by a comorbid condition; 3) persists even after effective treatment for the comorbid condition; and 4) exacerbates the symptoms of the comorbid condition (Riemann et al., 2015). Previous systematic reviews and/or meta-analyses investigated the effects of exercise on sleep on people with sleep complaints or chronic insomnia, undefined populations, and patients with sleep problems (Kubitz et al., 1996; Youngstedt, O'Connor & Dishman, 1997; Montgomery & Dennis, 2002; Montgomery & Dennis, 2004; Passos et al., 2012; Yang et al., 2012; Kredlow et al., 2015). A previous review also examined the effects of exercise on sleep for specific subpopulations (e.g., cancer survivors) (Mercier, Savard & Bernard, 2017). However, no previous systematic reviews have examined the effect of exercise on patients with primary and secondary insomnia as defined by having both sleep disruption and daytime impairment. Investigating the effect of exercise on patients with primary and secondary insomnia would be beneficial in clinical practice since the DSM-5 and ICSD-3 abolished the distinction between primary and secondary insomnia.

This review aimed to examine the efficacy of exercise among patients with insomnia.

**2. The contribution that the meta-analysis makes to knowledge in light of previously published related reports, including other meta-analyses and systematic reviews.**

We first performed a systematic review and meta-analysis of the effects of exercise on sleep in patients with insomnia (diagnosed by criteria or screened by questionnaires). Our findings suggest that the effects of exercise on sleep were larger in patients with insomnia than in other populations and should be an effective nonpharmacological intervention. Exercise interventions may alleviate symptoms in patients with insomnia without use of hypnotics. The American Academy of Sleep Medicine report does not include exercise as a viable recommendation for treating insomnia (Morgenthaler et al., 2006). Our findings suggest that future clinical practice guidelines should include exercise as recommendation for treating patients with insomnia.

**REFERENCES**

Chennaoui M, Arnal PJ, Sauvet F, Leger D. 2015. Sleep and exercise: a reciprocal issue? *Sleep Medicine Reviews* 20:59-72. 10.1016/j.smrv.2014.06.008

Driver HS, Taylor SR. 2000. Exercise and sleep. *Sleep Medicine Reviews* 4:387-402. 10.1053/smrv.2000.0110

Hartescu I, Morgan K, Stevinson CD. 2015. Increased physical activity improves sleep and mood outcomes in inactive people with insomnia: a randomized controlled trial. *Journal of Sleep Research* 24:526-534. 10.1111/jsr.12297

Kredlow MA, Capozzoli MC, Hearon BA, Calkins AW, Otto MW. 2015. The effects of physical activity on sleep: a meta-analytic review. *Journal of Behavioral Medicine* 38:427-449. 10.1007/s10865-015-9617-6

Kubitz KA, Landers DM, Petruzzello SJ, Han M. 1996. The effects of acute and chronic exercise on sleep. A meta-analytic review. *Sports Medicine* 21:277-291.

Mercier J, Savard J, Bernard P. 2017. Exercise interventions to improve sleep in cancer patients: A systematic review and meta-analysis. *Sleep Medicine Reviews* 36:43-56. 10.1016/j.smrv.2016.11.001

Montgomery P, Dennis J. 2002. Physical exercise for sleep problems in adults aged 60+. *Cochrane Database of Systematic Reviews*:CD003404. 10.1002/14651858.CD003404

Montgomery P, Dennis J. 2004. A systematic review of non-pharmacological therapies for sleep problems in later life. *Sleep Medicine Reviews* 8:47-62. 10.1016/S1087-0792(03)00026-1

Morgenthaler T, Kramer M, Alessi C, Friedman L, Boehlecke B, Brown T, Coleman J, Kapur V, Lee-Chiong T, Owens J, Pancer J, Swick T, American Academy of Sleep Medicine. 2006. Practice parameters for the psychological and behavioral treatment of insomnia: an update. An American Academy of Sleep Medicine report. *Sleep* 29:1415-1419.

Passos GS, Poyares D, Santana MG, Garbuio SA, Tufik S, Mello MT. 2010. Effect of acute physical exercise on patients with chronic primary insomnia. *Journal of Clinical Sleep Medicine* 6:270-275.

Passos GS, Poyares DL, Santana MG, Tufik S, Mello MT. 2012. Is exercise an alternative treatment for chronic insomnia? *Clinics* 67:653-660.

Reid KJ, Baron KG, Lu B, Naylor E, Wolfe L, Zee PC. 2010. Aerobic exercise improves self-reported sleep and quality of life in older adults with insomnia. *Sleep Medicine* 11:934-940. 10.1016/j.sleep.2010.04.014

Riemann D, Nissen C, Palagini L, Otte A, Perlis ML, Spiegelhalder K. 2015. The neurobiology, investigation, and treatment of chronic insomnia. *Lancet Neurology* 14:547-558. 10.1016/S1474-4422(15)00021-6

Yang PY, Ho KH, Chen HC, Chien MY. 2012. Exercise training improves sleep quality in middle-aged and older adults with sleep problems: a systematic review. *Journal of Physiotherapy* 58:157-163. 10.1016/S1836-9553(12)70106-6

Youngstedt SD. 2005. Effects of exercise on sleep. *Clinics in Sports Medicine* 24:355-365, xi. 10.1016/j.csm.2004.12.003

Youngstedt SD, O'Connor PJ, Dishman RK. 1997. The effects of acute exercise on sleep: a quantitative synthesis. *Sleep* 20:203-214.
