# Supplementary material for: Exercise can improve sleep quality: a systematic review and meta-analysis
Source: PeerJ. 2018 Jul 11;6:e5172. doi: 10.7717/peerj.5172 (PMC6045928; doi:10.7717/peerj.5172)
Supplement: Appendix S1 [file peerj-06-5172-s027.docx]

**Appendix S1: Search strategy**

**CENTRAL**

#1 MeSH descriptor: [Sleep Wake Disorders] explode all trees

#2 MeSH descriptor: [Dyssomnias] explode all trees

#3 #1 OR #2

#4 (insomnia or insomnias or sleepless):ti,ab,kw

#5 #3 OR #4

#6 MeSH descriptor: [Physical Exertion] explode all trees

#7 MeSH descriptor: [sports] explode all trees

#8 MeSH descriptor: [Exercise Therapy] explode all trees

#9 MeSH descriptor: [EXERCISE TEST] explode all trees

#10 MeSH descriptor: [Physical Endurance] explode all trees

#11 MeSH descriptor: [Motor Activity] explode all trees

#12 #6 OR #7 OR #8 OR #9 OR #10 OR #11

#13 (exercise or "physical activity" or exertion or "physical fitness" or walking or running or swimming or jogging or yoga or sports):ti,ab,kw

#14 #12 OR #13

#15 #5 AND #14

**MEDLINE (via EBSCO)**

#1 MH "Sleep Wake Disorders+"

#2 MH "Dyssomnias+"

#3 #1 or #2

#4 (MP insomn* or MP insomnia* or MP sleepless* or MP somnambul*)

#5 #3 or #4

#6 MH "physical exertion+"

#7 MH "sports+"

#8 MH "EXERCISE THERAPY+"

#9 MH "EXERCISE TEST+"

#10 MH "Physical Endurance+"

#11 MH "Motor Activity+"

#12 #6 or #7 or #8 or #9 or #10 or #11

#13 (TI exertion or TI walking or TI running or TI swimming or TI jogging or TI yoga or TI sports or AB exertion or AB walking or AB running or AB swimming or AB jogging or AB yoga or AB sports) or (MP exercise or MP exercis* or MP motor activit* or MP leisure activit* or MP physical fitness or MP physical endurance or MP exercise tolerance or MP aerobic or MP physical activity or MP physical capacity or MP physical performance training)

#14 #12 or #13

#15 (PT randomised controlled trial or PT controlled clinical trial) or AB randomized or AB placebo or MH "clinical trials as topic" or AB randomly or TI trial

#16 MH "animals+" not MH "humans"

#17 #15 not #16

#18 #5 and #14 and #17

**EMBASE (Via Elsevier)**

#1 ‘sleep disorder’/exp

#2 ‘insomnia’/exp

#3 ‘sleep deprivation’/exp

#4 #1 or #2 or #3

#5 ‘sleepless*’:ab,ti or ‘insomn*’:ab,ti or ‘somnambul*’:ab,ti

#6 #4 or #5

#7 ‘physical education’/exp

#8 ‘fitness’/exp

#9 ‘sport’/exp

#10 ‘kinesiotherapy’/exp

#11 ‘exercise tolerance’/exp

#12 ‘endurance’/exp

#13 ‘motor activity’/exp

#14 #7 or #8 or #9 or #10 or #11 or #12 or #13

#15 ‘exertion’:ab,ti or ‘walking’:ab,ti or ‘running’:ab,ti or ‘swimming’:ab,ti or ‘jogging’:ab,ti or ‘yoga’:ab,ti or ‘sport*’:ab,ti or ‘exercise*’:ab,ti or ‘exercis*’:ab,ti or ‘motor activit*’:ab,ti or ‘leisure activit*’:ab,ti or ‘physical fitness’:ab,ti or ‘physical endurance’:ab,ti or ‘exercise tolerance*’:ab,ti or ‘aerobic*’:ab,ti or ‘physical activit*’:ab,ti or ‘physical capacity*’:ab,ti or ‘physical performance*’:ab,ti

#16 #14 or #15

#17 random*:ab,ti or placebo*:de,ab,ti or (double NEXT/1 blind*):ab,ti

#18 #6 and #16 and #17

**PsycINFO (via PsycNet)**

#1 Index Terms: {Hypersomnia} OR Index Terms: {Insomnia} OR Index Terms: {Kleine Levin Syndrome} OR Index Terms: {Narcolepsy} OR Index Terms: {Parasomnias} OR Index Terms: {Sleep Deprivation} OR Index Terms: {Sleep Disorders} OR Index Terms: {Sleepwalking}

#2 Abstract: sleepless* OR Title: sleepless* OR Abstract: insomn* OR Title: insomn* OR Abstract: somnambul* OR Title: somnambul*

#3 #1 OR #2

#4 Index Terms: {Actigraphy} OR Index Terms: {Exercise} OR Index Terms: {Physical Activity}

#5 Index Terms: {Physical Education}

#6 Index Terms: {Physical Fitness}

#7 Index Terms: {Baseball} OR Index Terms: {Basketball} OR Index Terms: {Extreme Sports} OR Index Terms: {Football} OR Index Terms: {Judo} OR Index Terms: {Martial Arts} OR Index Terms: {Soccer} OR Index Terms: {Sports} OR Index Terms: {Swimming} OR Index Terms: {Tennis} OR Index Terms: {Weightlifting}

#8 Index Terms: {Movement Therapy}

#9 Index Terms: {Physical Endurance}

#10 Index Terms: {Finger Tapping} OR Index Terms: {Jumping} OR Index Terms: {Motor Performance} OR Index Terms: {Running} OR Index Terms: {Walking}

#11 #4 OR #5 OR #6 OR #7 OR #8 OR #9 OR #10

#12 Abstract: exertion OR Title: exertion OR Abstract: walking OR Title: walking OR Abstract: running OR Title: running OR Abstract: swimming OR Title: swimming OR Abstract: jogging OR Title: jogging OR Abstract: yoga OR Title: yoga OR Abstract: sport* OR Title: sport* OR Abstract: exercise* OR Title: exercise* OR Abstract: exercis* OR Title: exercis* OR Abstract: "motor activit*" OR Title: "motor activit*" OR Abstract: "leisure activit*" OR Title: "leisure activit*" OR Abstract: "physical fitness" OR Title: "physical fitness" OR Abstract: "physical endurance" OR Title: "physical endurance" OR Abstract: "exercise tolerance*" OR Title: "exercise tolerance*" OR Abstract: aerobic* OR Title: aerobic* OR Abstract: "physical activit*" OR Title: "physical activit*" OR Abstract: "physical capacity*" OR Title: "physical capacity*" OR Abstract: "physical performance*" OR Title: "physical performance*"

#13 #11 OR #12

#14 Any Field: "double-blind" OR Any Field: "random* assigned" OR Any Field: control

#15 #3 AND #13 AND #14

**clinicaltrials.gov**

Advanced search

#1 Conditions: (“sleep initiation and maintenance disorders” OR sleep wake disorders OR sleep deprivation OR insomnia OR insomniac OR sleepless OR dyssomnia)

#2 Interventions: (exertion OR walking OR running OR swimming OR jogging OR yoga OR sports OR exercise OR physical fitness OR physical endurance OR aerobic OR physical activity OR physical performance training OR “physical education and training” OR exercise therapy)

#3 #1 AND #2

**ICTRP**

Advanced search

#1 Condition: (sleep initiation and maintenance disorders OR sleep wake disorders OR sleep deprivation OR insomnia* OR sleepless OR dyssomnia*)

#2 Intervention: (exertion OR walking OR running OR swimming OR jogging OR yoga OR sports OR exercis* OR physical fitness OR physical endurance OR aerobic OR physical activity OR physical performance training OR physical education and training OR exercise therapy)

#3 #1 AND #2

Recruitment status is ALL.

We performed electric search using the following filters; Cochrane Highly Sensitive Search Strategy for identifying randomized trials in MEDLINE: sensitivity- and precision-maximizing version (2008 revision); Ovid format in Ovid (Higgins & Green, 2011), best optimization of sensitivity and specificity (Alternative Embase strategies amended to embase.com format) in EMBASE (Wong, Wilczynski & Haynes, 2006), best optimization of sensitivity and specificity (Strategies for finding methodologically sound therapy studies in PsycInfo1, amended to ProQuest format) in PsycINFO (Eady, Wilczynski & Haynes, 2008).

**REFERENCES**

Eady AM, Wilczynski NL, Haynes RB. 2008. PsycINFO search strategies identified methodologically sound therapy studies and review articles for use by clinicians and researchers. *Journal of Clinical Epidemiology* 61:34-40. 10.1016/j.jclinepi.2006.09.016

Higgins J, Green S. 2011. Cochrane Handbook for Systematic Reviews of Interventions Version 5.1.0 [updated March 2011]. *Available at* [*http://handbook-5-1.cochrane.org/*](http://handbook-5-1.cochrane.org/) (accessed 2 June 2018).

Wong SS, Wilczynski NL, Haynes RB. 2006. Developing optimal search strategies for detecting clinically sound treatment studies in EMBASE. *Journal of the Medical Library Association* 94:41-47.
