# Supplementary material for: Exercise can improve sleep quality: a systematic review and meta-analysis
Source: PeerJ. 2018 Jul 11;6:e5172. doi: 10.7717/peerj.5172 (PMC6045928; doi:10.7717/peerj.5172)
Supplement: Appendix S2 [file peerj-06-5172-s028.docx]

**Appendix S2: Information extracted from the included studies**

1) General information

Author, Year of publication, Title, Journal (title, volume, pages), Language, Country, Publication status, Country, Funding sources

2) Trial design

Design (parallel and others), Randomization (Individual, cluster and unclear), Blindness, Date of study initiation

3) Participants

Patient status (primary insomnia, secondary insomnia, primary and secondary insomnia), Insomnia diagnosis (DSM, ICSD, RDC, predetermined criteria), Total sample size, Age, Sex, Setting

4) Intervention

Number in experimental group; Number in control group; Term of exercise; Frequency of exercise; Duration of exercise: short-term (<2 mo), medium-term (2 to <6 mo), long-term (>6 mo); Intensity of exercise: aerobic or anaerobic; Type of exercise: aerobic (walking and other exercises), resistance, aerobic and resistance; Setting or location of exercise: home, physical therapy center, hospital, elsewhere; Nature of control group (e.g. No intervention or sleep hygiene education, standard care)

5) Outcomes

Methods of assessment; Number of missing patients; Pre-test and post-test means or change scores and standard deviations for all groups for all outcomes specified above; Follow-up period
