# Supplementary figures and images for: Exercise can improve sleep quality: a systematic review and meta-analysis
Source: PeerJ. 2018 Jul 11;6:e5172. doi: 10.7717/peerj.5172 (PMC6045928; doi:10.7717/peerj.5172)

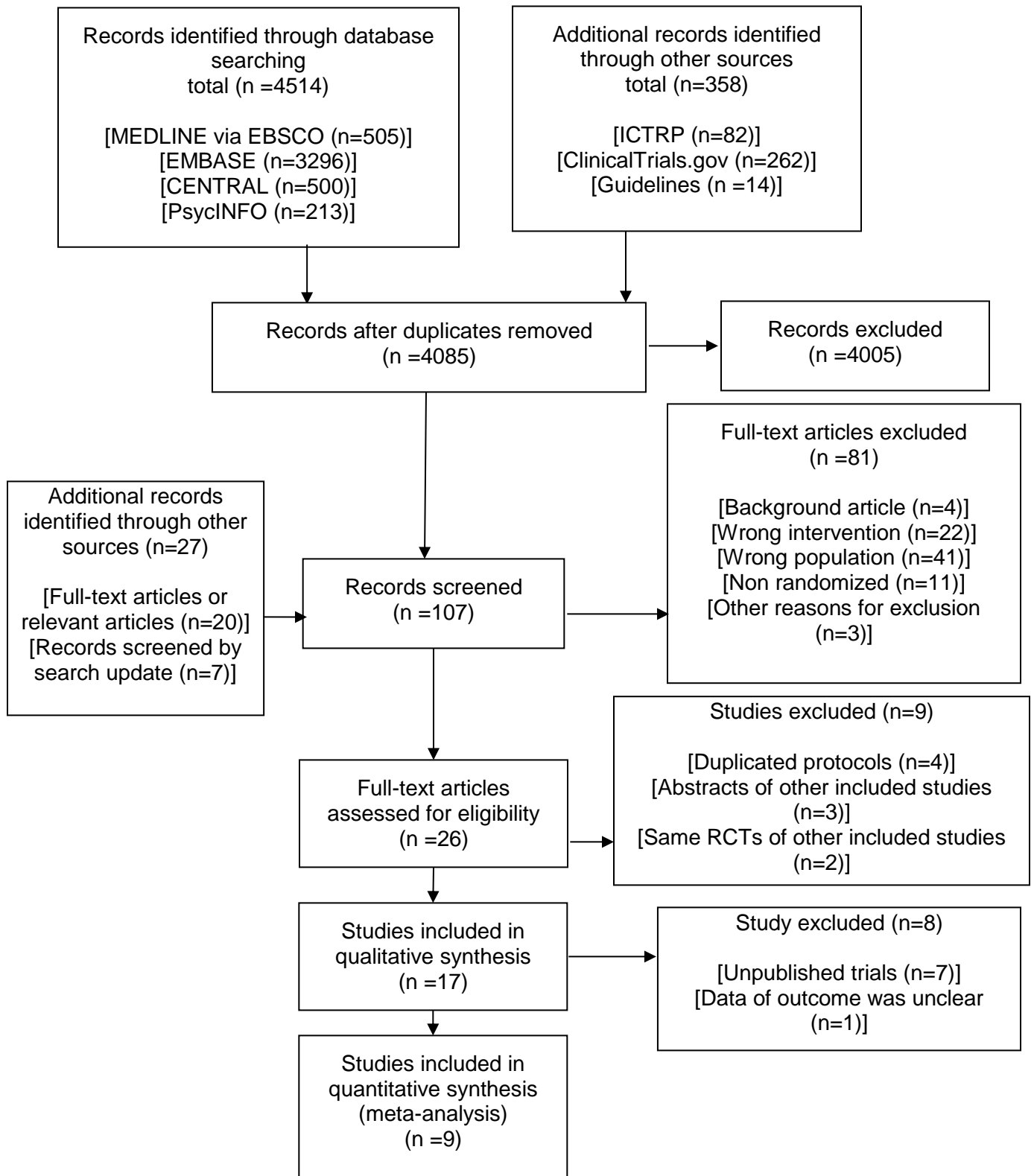

Supplement: Supplemental Information 1 [file peerj-06-5172-s030.pdf]
